# Phylogeography of hydrothermal vent stalked barnacles: a new species fills a gap in the Indian Ocean ‘dispersal corridor’ hypothesis

**DOI:** 10.1098/rsos.172408

**Published:** 2018-04-18

**Authors:** Hiromi Kayama Watanabe, Chong Chen, Daniel P. Marie, Ken Takai, Katsunori Fujikura, Benny K. K. Chan

**Affiliations:** 1 Department of Marine Biodiversity Research, Japan Agency for Marine-Earth Science and Technology, Yokosuka, Kanagawa, Japan; 2 Department of Subsurface Geobiological Research, Japan Agency for Marine-Earth Science and Technology, Yokosuka, Kanagawa, Japan; 3 Mauritius Oceanography Institute, Avenue des Anchois, Morcellement de Chazal, Albion, Mauritius; 4 Biodiversity Research Center, Academia Sinica, Taipei 115, Taiwan, Republic of China

**Keywords:** chemosynthesis-based ecosystem, Eolepadidae, *Neolepas*

## Abstract

Phylogeography of animals provides clues to processes governing their evolution and diversification. The Indian Ocean has been hypothesized as a ‘dispersal corridor’ connecting hydrothermal vent fauna of Atlantic and Pacific oceans. Stalked barnacles of the family Eolepadidae are common associates of deep-sea vents in Southern, Pacific and Indian oceans, and the family is an ideal group for testing this hypothesis. Here, we describe *Neolepas marisindica* sp. nov. from the Indian Ocean, distinguished from *N. zevinae* and *N. rapanuii* by having a tridentoid mandible in which the second tooth lacks small elongated teeth. Morphological variations suggest that environmental differences result in phenotypic plasticity in the capitulum and scales on the peduncle in eolepadids. We suggest that diagnostic characters in Eolepadidae should be based mainly on more reliable arthropodal characters and DNA barcoding, while the plate arrangement should be used carefully with their intraspecific variation in mind. We show morphologically that *Neolepas* specimens collected from the South West Indian Ridge, the South East Indian Ridge and the Central Indian Ridge belong to the new species. Molecular phylogeny and fossil evidence indicated that *Neolepas* migrated from the southern Pacific to the Indian Ocean through the Southern Ocean, providing key evidence against the ‘dispersal corridor’ hypothesis. Exploration of the South East Indian Ridge is urgently required to understand vent biogeography in the Indian Ocean.

## 1. Introduction

Distribution range and phylogeography of organisms are important basic ecological traits for elucidating their evolutionary history and their successful conservation. Distribution ranges of deep-sea animals are poorly understood, with the exception of those associated with hydrothermal vents which have been relatively well studied (e.g. [1,2]). Historical migrations across a geological timescale, species distributional ranges and biogeographical provinces of hydrothermal vent animals have been discussed on the basis of faunal compositions in local communities [1–5], molecular phylogenetic analysis (e.g. [6,7]) and recently, physico-oceanographic modelling (e.g. [8]). These studies revealed the crucial influence of plate tectonics, geological structures and oceanic current systems to realized ranges of metapopulations, as well as the biogeography of vent animals.

The Indian Ocean hosts three oceanic ridges: the Central Indian Ridge (CIR), the South West Indian Ridge (SWIR) and the South East Indian Ridge (SEIR). These ridges were suggested to act as corridors of dispersal for vent animals between Atlantic and Pacific oceans [9]. Hydrothermal activities in the Indian Ocean were first detected on the SEIR, with vertical profiles of thermometer and nephelometer equipped on dredges and core samplers detecting hydrothermal plumes at ‘site 21’ near the Amsterdam--St Paul Plateau, and the dredge successfully collected a new species of vent-associated barnacle belonging to the genus *Neolepas* [10]. Morphological characteristics of this vent barnacle from the SEIR were given [11,12], but without a name or formal description. As then, although both CIR and SWIR have been explored by manned submersibles and remotely operated vehicles [13–16], hydrothermal vents on the SEIR have never been observed directly. The distribution ranges of hydrothermal fauna across the entire Indian Ocean ridge systems, therefore, have not been elucidated in its entirety.

Barnacles of the family Eolepadidae, which includes the genus *Neolepas*, have been widely reported from deep-sea chemosynthetic environments in the Indo-Pacific and Southern oceans (from East Scotia Ridge, which is in the South Atlantic) [17], but they are apparently absent from the central and northern Atlantic Ocean and also the Arctic Ocean [18,19]. The first eolepadid barnacle to receive a formal description, *Neolepas zevinae* Newman 1979 [20] was collected at 21° N on the East Pacific Rise (EPR), off Mexico. At that time, *Neolepas* was classified in the subfamily Lithotryinae under Scalpellidae, based on having eight capitular plates. This genus was later transferred to a new subfamily, Eolepadinae, under Scalpellidae [21]. The second species in the genus, *Neolepas rapanuii* Jones 1993 [22] was identified and described from the 23° S site on the EPR, off Easter Island. Subsequently, Eolepadinae was elevated to a full family, Eolepadidae. A new subfamily, Neolepadinae, was established for *Neolepas* and the other subfamily, Eolepadinae, currently only houses two fossil genera—*Archaeolepas* and *Eolepas* [23]. The third *Neolepas* species, *Neolepas osheai* Buckeridge 2000 [13], was described from the South West Pacific [24]; but was later transferred to a new genus, *Vulcanolepas*, in the light of the discovery of another new genus and species, *Leucolepas longa* Southward & Jones 2003 in Edison Seamount [12]. Additionally, a fossil species that probably belongs to *Neolepas*, *?Neolepas augurata* Buckeridge & Grant-Mackie 1985 [25] has been recorded from the lower Jurassic of New Caledonia. *Leucolepas* remains monotypic to date, while *Vulcanolepas* now further includes *Vulcanolepas parensis* Southward 2005 [26] from the Pacific-Antarctic Ridge and *Vulcanolepas scotiaensis* Buckeridge & Linse 2013 [27] from the East Scotia Ridge (ESR), the Southern Ocean. Another species of *Vulcanolepas* has been found in Lau Basin vents in the Western Pacific [6], which is currently under description (BKK Chan 2018, personal communication). A final genus currently included in Neolepadinae is *Ashinkailepas*, with two species in the Western Pacific (*Ashinkailepas seepiophila* Yamaguchi, Newman & Hashimoto 2004 [28] and *Ashinkailepas kermadecensis* Buckeridge 2009 [29]) [30].

Despite these progresses in the systematics of Eolepadidae, several *Neolepas* populations in the Indian Ocean, including the dredged specimens from SEIR and populations from CIR and SWIR, remain undescribed [17,31]. Molecular phylogenetics of Eolepadidae showed that *Neolepas* populations from the CIR and the SWIR exhibited distinct sequence divergence from other described eolepadid species [6], indicating that the Indian Ocean taxa indeed represent an undescribed species (*Neolepas* sp. 1 *sensu* Herrera *et al*. [6]). This study aims to characterize and describe this new *Neolepas* species mainly using material from CIR but supported by evidence from SWIR and SEIR to consider its distributional range across all three oceanic ridges in the Indian Ocean, as well as the global phylogeography of living eolepadid barnacles.

## 2. Material and methods

### 2.1. Sampling sites

**Figure 1.**
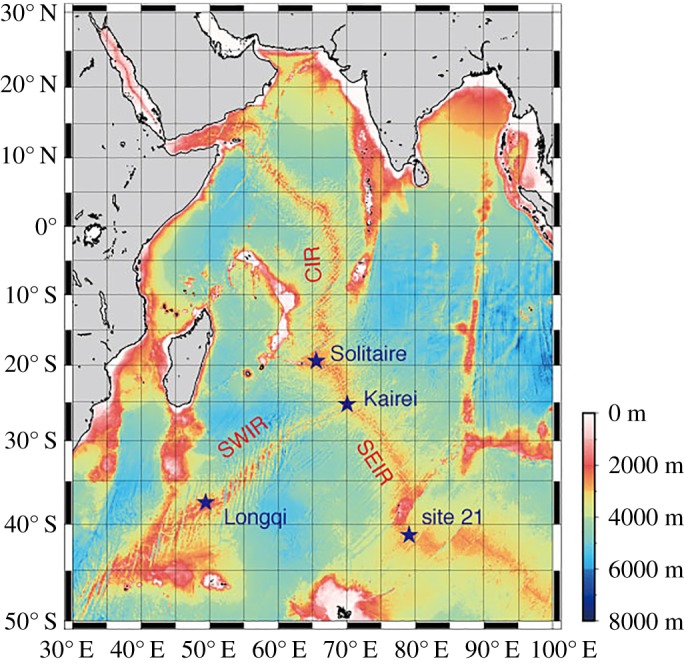
Map of Indian Ocean Ridges, showing the locations of the four hydrothermal vent fields; Kairei and Solitaire fields on the Central Indian Ridge, Longqi field on the South West Indian Ridge, and site 21 near Amsterdam--St Paul Plateau on the South East Indian Ridge.

**Figure 2.**
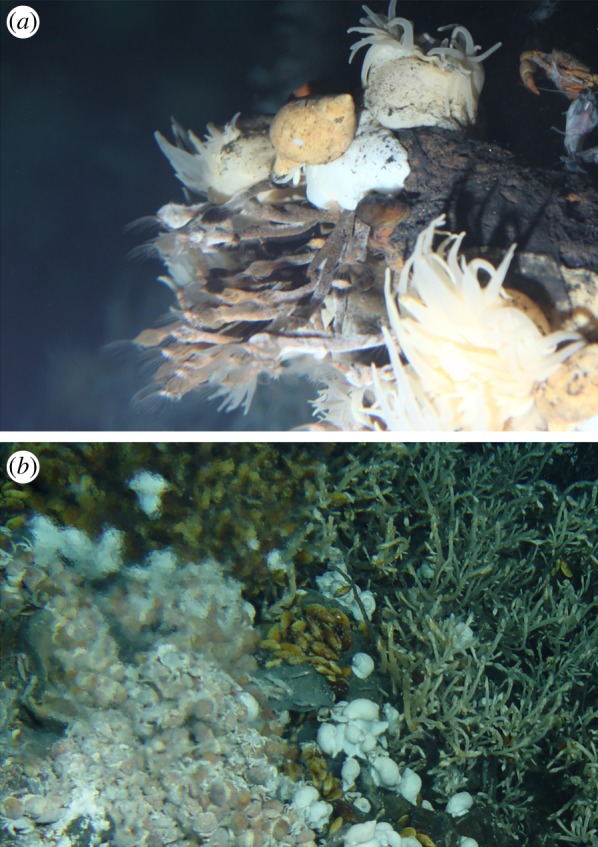
Habitats of *Neolepas marisindica* sp. nov. (*a*) Kairei hydrothermal vent field, CIR, (*b*) Solitaire hydrothermal vent field, CIR.

Eolepadid stalked barnacles were collected from Kairei and Solitaire hydrothermal vent fields on CIR using the Human Occupied Vehicle (HOV) *Shinkai 6500* on-board R/V *Yokosuka* of Japan Agency for Marine-Earth Science and Technology (JAMSTEC), during research cruises YK09-13, YK13-02 and YK16-E02 (figures 1 and 2; for YK09-13 also see [14]).

### 2.2. Morphological examination

The barnacles were dissected and the body, including six pairs of cirri, the oral cone, the caudal appendages and the penis, were examined by light microscopy (Zeiss Axio-scope and stereomicroscope Leica M80). The terminology used to describe eolepadid barnacles herein follows those in the previous studies [12,27], whereas the setal classification and description follow the more general terminology for barnacles overall [32]. Type and voucher specimens were deposited in the National Museum of Nature and Science, Tsukuba (NSMT) and the University Museum, the University of Tokyo (UMUT).

### 2.3. Comparison of capitular morphology between Kairei and Solitaire populations

To compare morphological differences between specimens taken from Kairei and Solitaire hydrothermal vent fields, the peduncle length, capitular height, height of rostrum and median latus, number of peduncular scales per whorl just below the capitulum region, width of scales (from three scales), size of scales projected from the peduncles (from three scales) were measured using a digital caliper (±0.1 mm). The angle of the tergal apex was measured from photographs showing the lateral view of the capitulum, using the image analysis software Sigma Scan Pro 5. For each specimen, the ratio of peduncle : capitulum length, the ratio of rostrum : median latus, the size of projecting scales and the tergal apex angle were obtained. Variation in each capitular character between the two populations was tested using either *t*-test or Wilcoxon Rank Sum test (when the normality assumption was violated).

### 2.4. Molecular phylogenetic analysis

Genomic DNA was extracted using DNeasy Blood & Tissue Kit (QIAGEN) from the adductor muscle of barnacle specimens. Partial sequence of the mitochondrial cytochrome *c* oxidase subunit I (COI) gene was amplified by polymerase chain reaction (PCR) using universal primer sets (LCO1490 and HCO2198, COI-3 and COI-6 [33,34]) and the Premix *ExTaq* Hot Start (TaKaRa). PCR was carried out in the following steps: initial denaturation at 94°C for 3 min and 35 cycles of denature (94°C for 30 s), annealing (50°C for 30 s) and extension (72°C for 90 s). PCR products were purified using Exo-SAP-it (USB, Affimetrix), following standard protocols. After BigDye reaction with BigDye Terminator v. 3.1, the products were sequenced using an ABI3130 automated sequencer (Applied Biosystems, Thermo Fisher). Electrophenograms obtained were checked by eye and assembled by Geneious v. 9 (Biomatters Limited) and registered to DNA Data Bank of Japan, with accession numbers LC350007–LC350015.

The sequences obtained were aligned with eolepadid sequences available in the databases of the International Nucleotide Sequence Database Collaboration, using Clustal X included in MEGA v. 6.06 [35]. A total of 123 sequences from seven eolepadid taxa were used (4--45 individuals per taxa), with one sequence of the pollicipedid barnacle *Capitulum mitella* (Linnaeus [36]) as the outgroup. Electronic supplementary material, table S1 shows the full list of sequences used in this study. The model selection programme in the same software was applied to select the best model for the maximum-likelihood algorithm, which was the Tamura three-parameter + Gamma distribution model. MEGA v. 6.06 was also used to reconstruct the phylogenetic trees using the maximum-likelihood algorithm, with 2000 bootstrap replicates.

## 3. Results

### 3.1. Systematics

Superorder Thoracica Darwin [37].

Order Scalpelliformes Buckeridge & Newman [38].

Family Eolepadidae Buckeridge [21]

Subfamily Neolepdinae Yamaguchi *et al*. [28]

Genus *Neolepas* Newman [20]

*Neolepas marisindica* sp. nov. Watanabe, Chen & Chan

Figures 3–12

**Figure 3.**
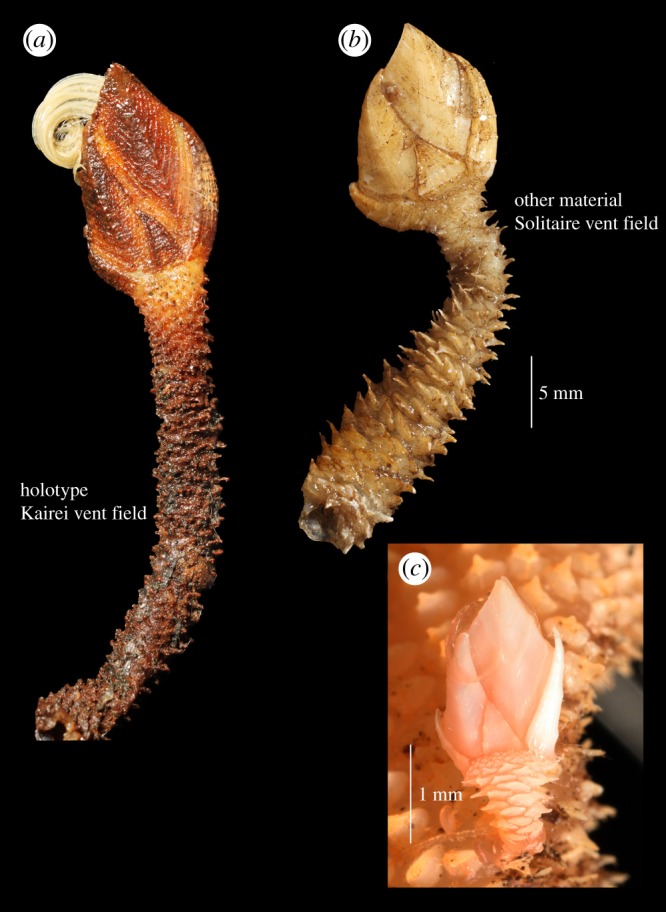
*Neolepas marisindica* sp. nov. (*a*) Holotype from Kairei vent field on the CIR (NSMT-Cr 26832). (*b*) A specimen collected from Solitaire vent field on the CIR (NSMT-Cr 26833). Note the variation in peduncular scales between the two populations. (*c*) A juvenile on the stalk of a specimen collected from the Solitaire vent field.

**Figure 4.**
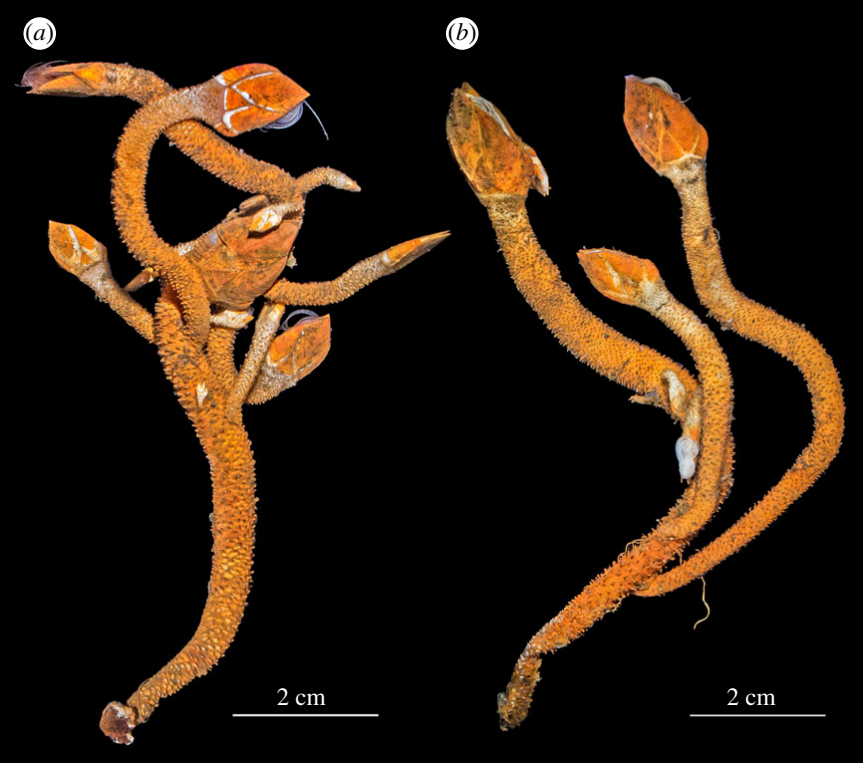
*Neolepas marisindica* sp. nov. (*a*) Paratype #1 (NSMT-Cr 26833), (*b*) paratype #2 (UMUT RA32760); both are from Kairei vent field on the CIR.

**Figure 5.**
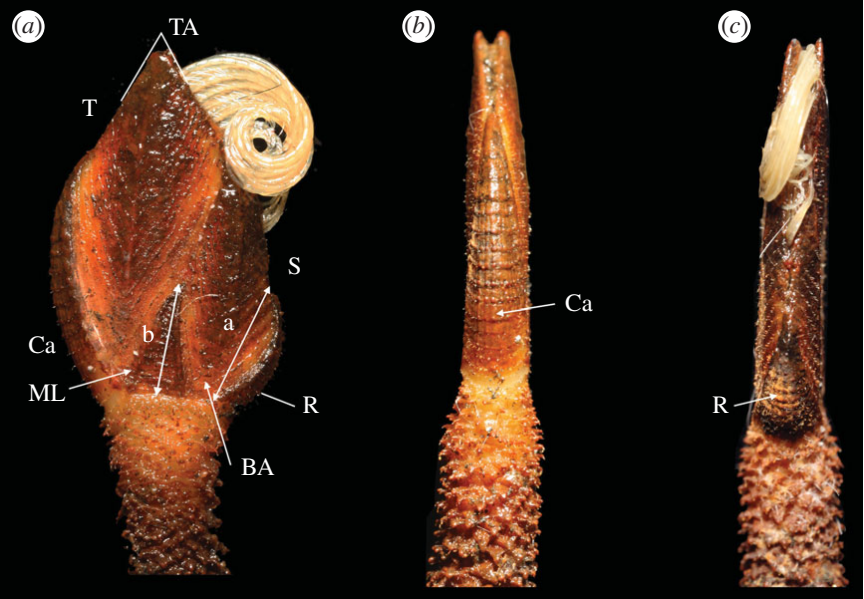
*Neolepas marisindica* sp. nov. Holotype (NSMT-Cr 26832), showing (*a*) lateral, (*b*) carinal and (*c*) rostral views of the capitulum. T, tergum; S, Scutum; Ca, Carina; R, Rostrum; BA, basal angle of tergum; TA, tergal apex angle; ML, median latus. Ratio of rostrum: carina is a/b.

**Figure 6.**
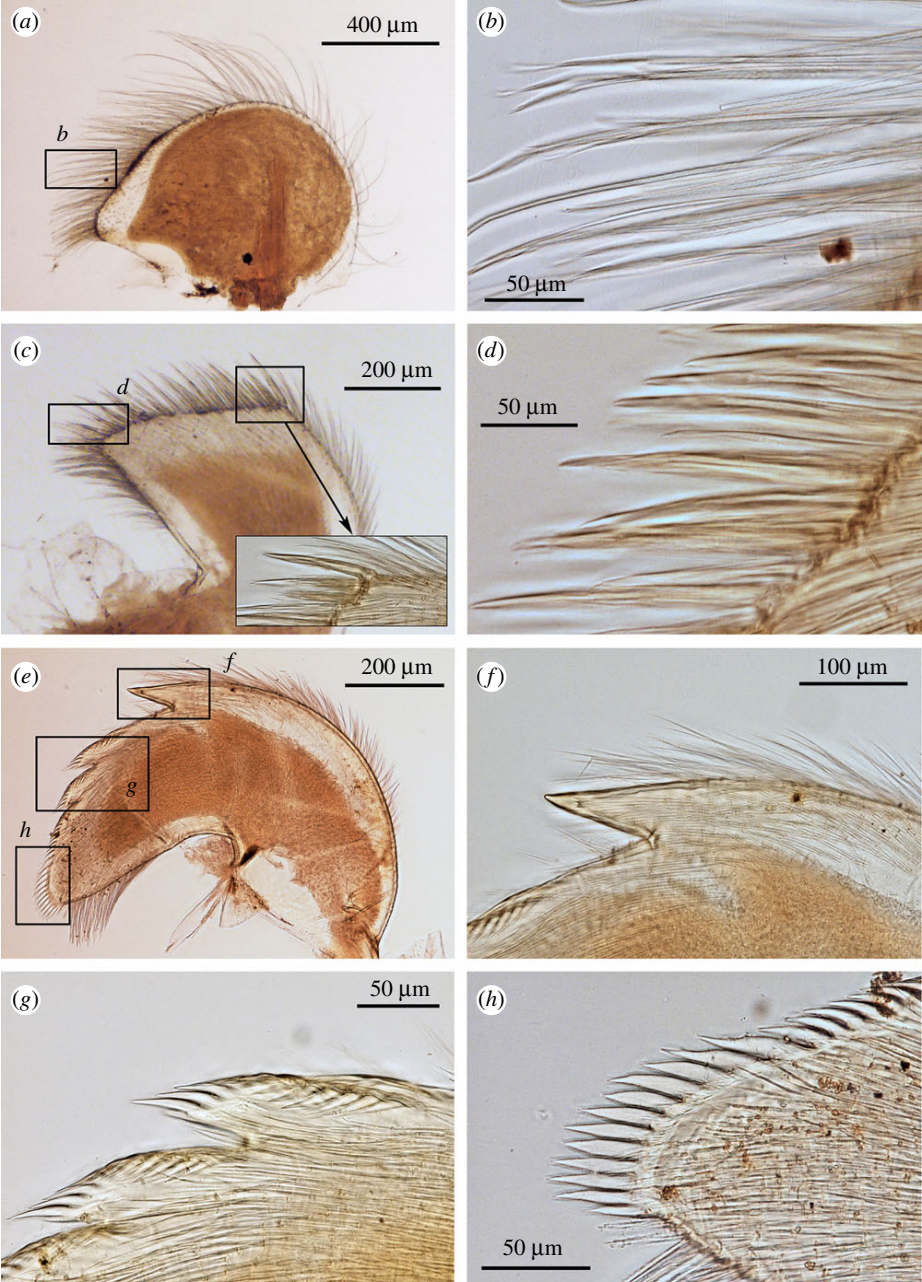
*Neolepas marisindica* sp. nov. Holotype (NSMT-Cr 26832). Oral cone. (*a*) Maxilla, (*b*) simple setae on the margin of maxilla, (*c*) maxillule, (*d*) spines on the cutting edge of maxillule, (*d*) spines on the cutting edge of maxillule, (*e*) mandibles (ventral view), (*f*) first tooth of mandible (ventral view), (*g*) second and third tooth (ventral view), (*h*) inferior angle of mandible (ventral view).

**Figure 7.**
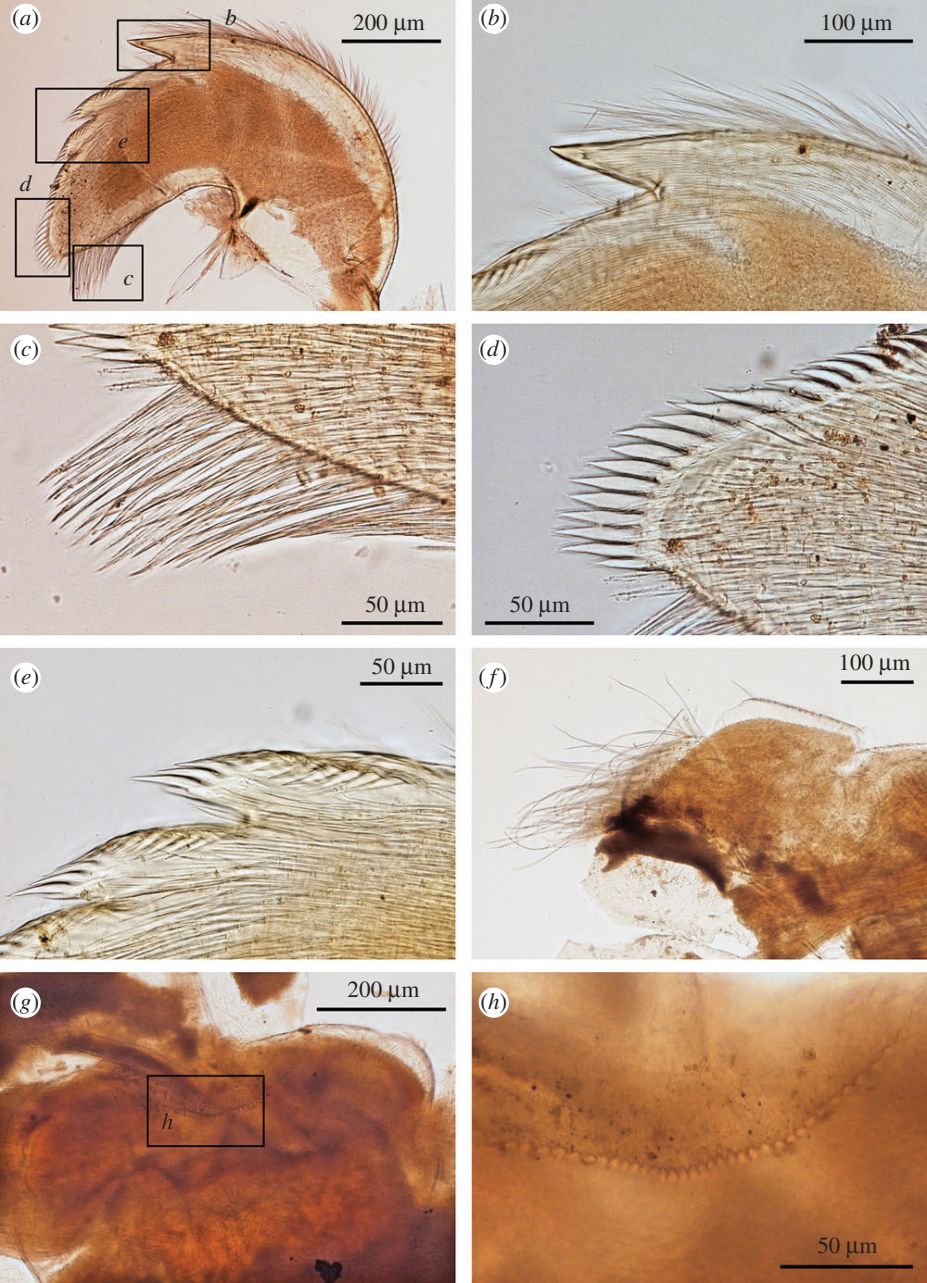
*Neolepas marisindica* sp. nov. Holotype (NSMT-Cr 26832). (*a*) Mandible (dorsal view), (*b*) second and third teeth (dorsal view), (*c*) lower margin (dorsal view), (*d*) inferior angle of mandible (dorsal view), (*e*) second and third tooth (dorsal view), (*f*) mandibulatory palp, (*g*) labrum, (*h*) cutting edge of labrum.

**Figure 8.**
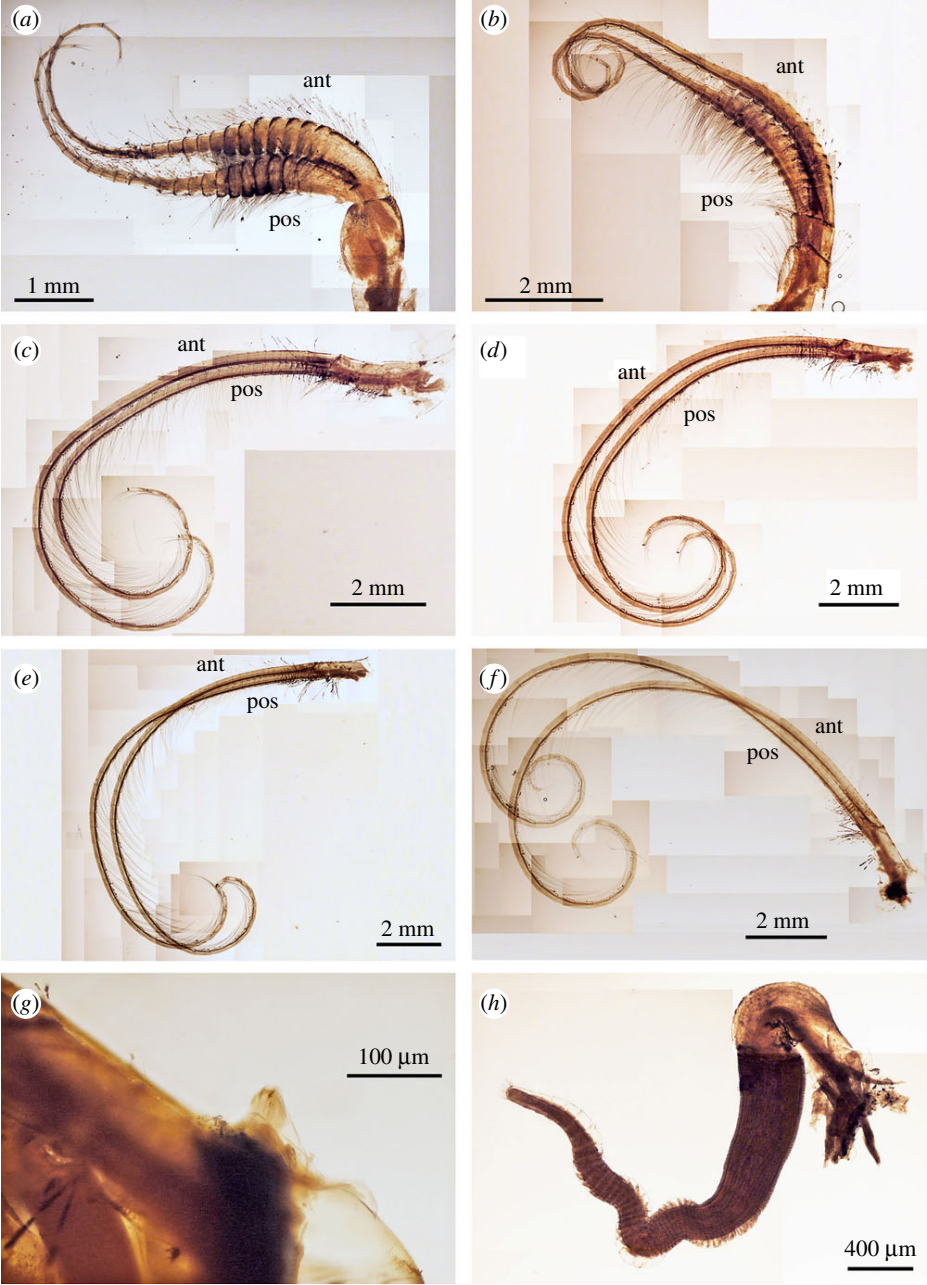
*Neolepas marisindica* sp. nov. Holotype (NSMT-Cr 26832). (*a*) Cirrus I, (*b*) cirrus II, (*c*) cirrus III, (*d*) cirrus IV, (*e*) cirrus V, (*f*) cirrus VI, (*g*) caudal appendages, (*h*) penis. ant, anterior ramus, pos, posterior ramus.

**Figure 9.**
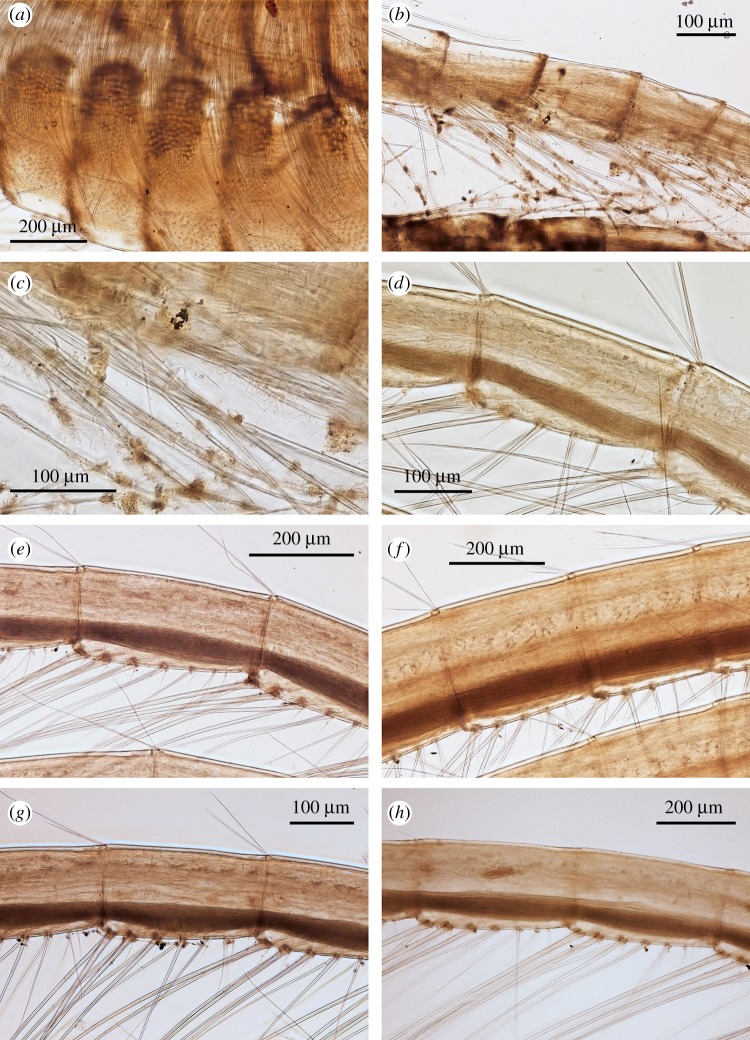
*Neolepas marisindica* sp. nov. Holotype (NSMT-Cr 26832). (*a*) Simple setae on proximal region of cirrus I, (*b*) simple setae on distal region of cirrus I, (*c*) simple setae on cirrus II, (*d*): intermediate segment of anterior ramus on cirrus II, (*e*) intermediate segment of anterior ramus on cirrus III, (*f*) intermediate segment of anterior ramus on cirrus IV, (*g*) intermediate segment of anterior ramus on cirrus V, (*h*) intermediate segment of anterior ramus on cirrus VI.

**Figure 10.**
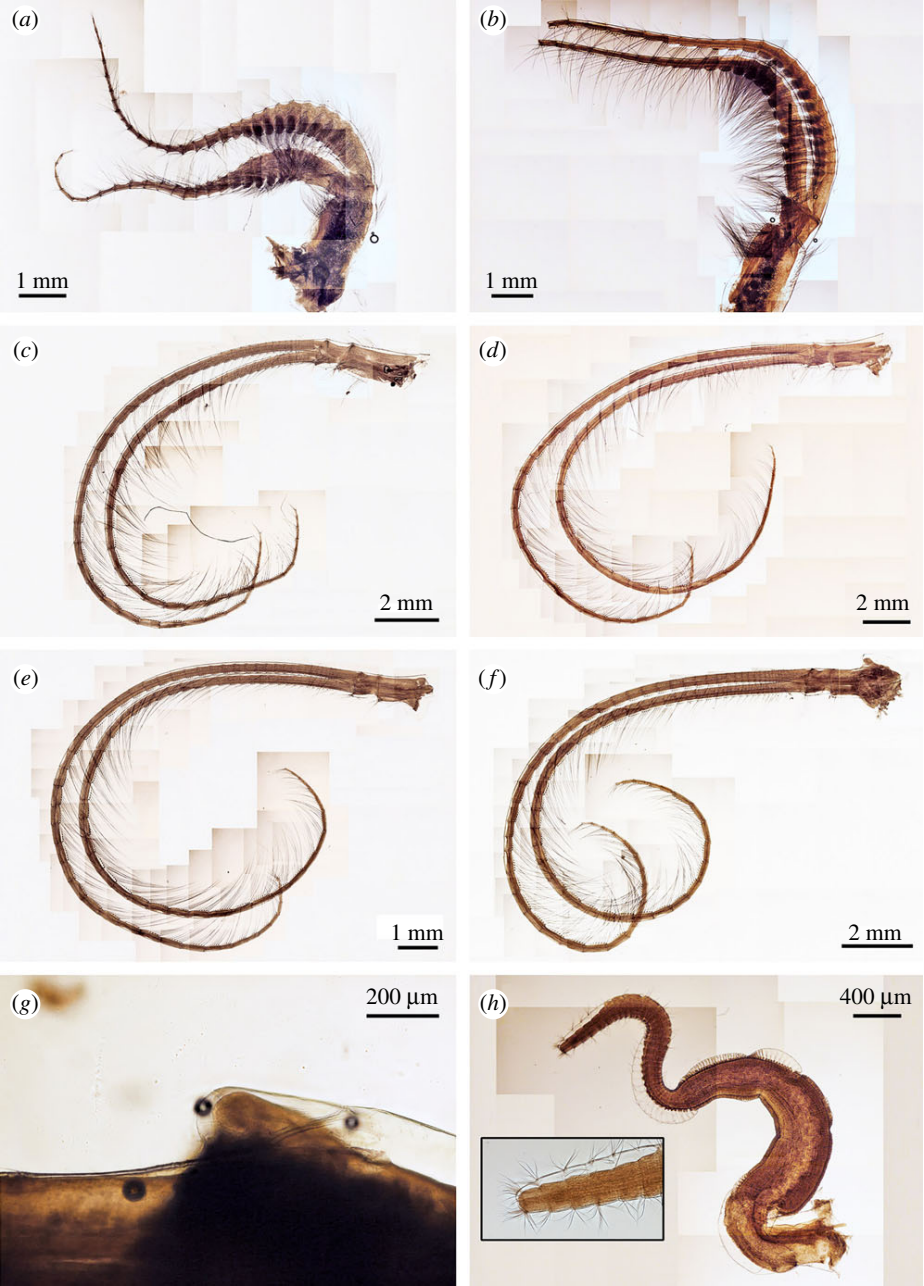
*Neolepas marisindica* sp. nov. A specimen collected from Solitaire vent field (NSMT-Cr 26834). (*a*) Cirrus I, (*b*) cirrus II, (*c*) cirrus III, (*d*) cirrus IV, (*e*) cirrus V, (*f*) cirrus VI, (*g*) caudal appendages, (*h*) penis.

**Figure 11.**
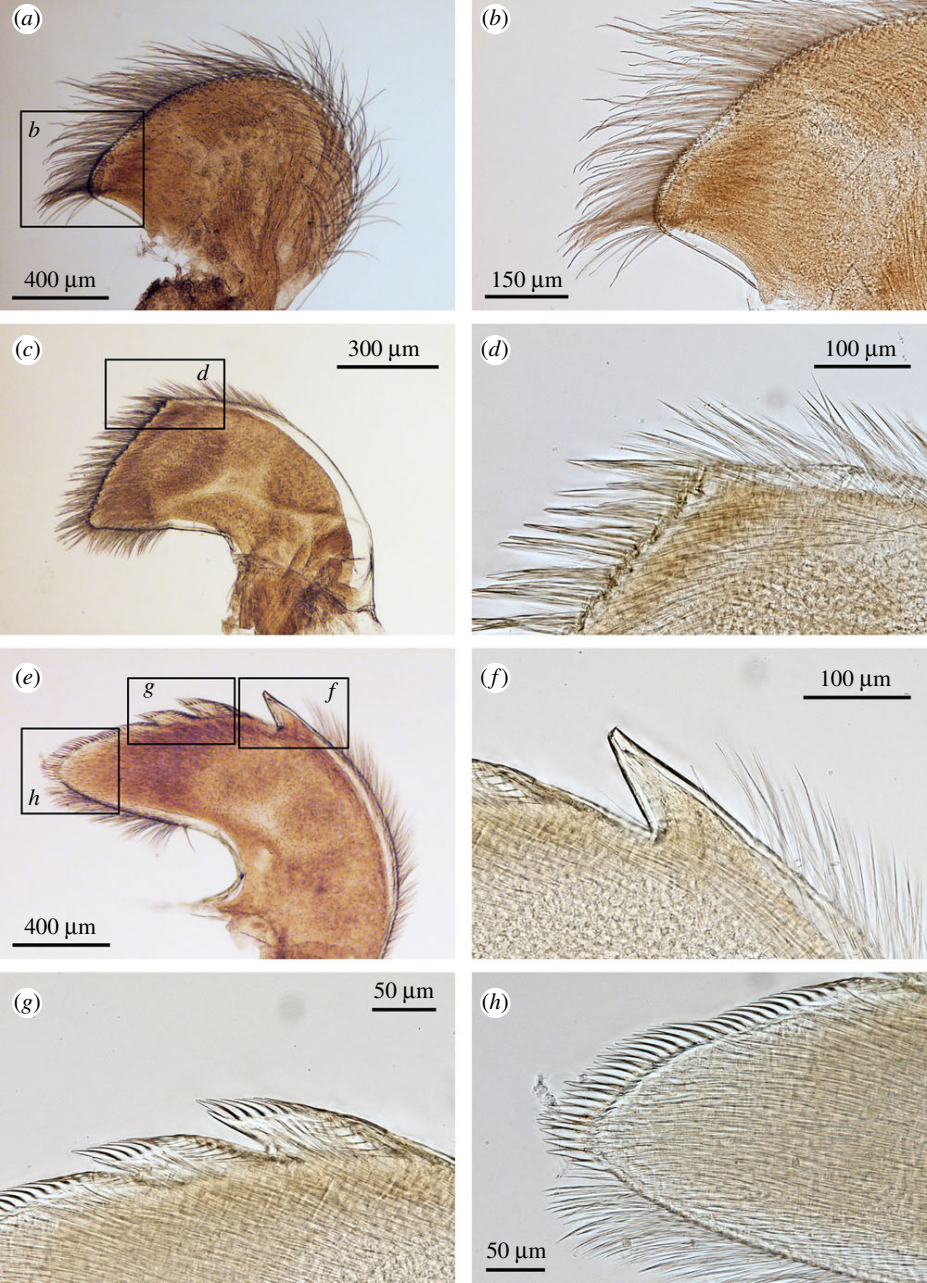
*Neolepas marisindica* sp. nov. A specimen from Solitaire vent field (NSMT-Cr 26834). Oral cone. (*a*) Maxilla, (*b*) simple setae on the margin of maxilla, (*c*) maxillule, (*d*) spines on the cutting edge of maxillule, (*e*) mandibles (ventral view), (*f*) first tooth of mandible (ventral view), (*g*) second and third tooth (ventral view), (*h*) inferior angle of mandible.

**Figure 12.**
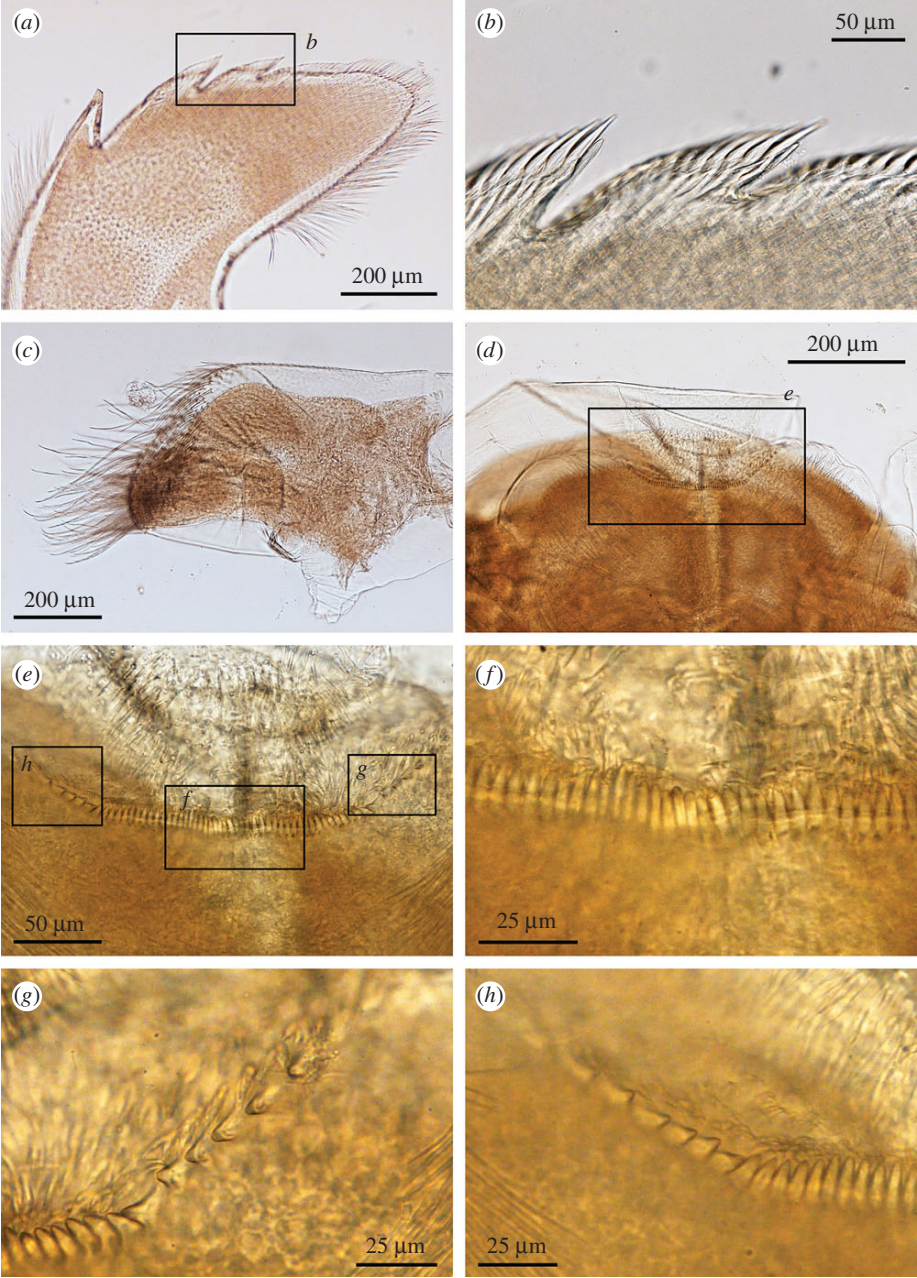
*Neolepas marisindica* sp. nov. A specimen collected from the Solitaire vent field (NSMT-Cr 26834). (*a*) Mandible (dorsal view), (*b*) second and third teeth (dorsal view), (*c*) mandibulatory palp, (*d*) labrum, (*e–h*) cutting edge of labrum.

Unnamed Indian Ocean Ridge species – Southward and Jones: 2003 [12], figs 18D, 19F, tables 7–9

*Neolepas* sp. – Hashimoto *et al.* [13], table 1

**Table 1.** *Neolepas marisindica* sp. nov. Segment counts of cirri on anterior and posterior ramus.

	I	II	III	IV	V	VI
Kairei	(holotype)					
anterior	29	30	43	45	51	50
posterior	25	29	39	45	49	48
Solitaire						
anterior	31	25	44	52	52	49
posterior	26	25	41	50	53	55

*Neolepas* n. sp. – Van Dover *et al*. [2], fig. 2*g*

*Leucolepas* sp. – Nakamura *et al.* [14], fig. 4 and table 1

‘*Neolepas* sp. 1. CIR Kairei’ – Herrera *et al*. [6], fig. 2; electronic supplementary material, table S1

‘*Neolepas* sp. 1. SWIR Dragon’ – Herrera *et al.* [6], fig. 2; electronic supplementary material, table S1

‘*Neolepas* sp. 1’ – Copley *et al*. [16], table 1

**ZooBank Registration.** http://zoobank.org/urn:lsid:zoobank.org:act:0C5ECE6C-DCF7-4647-92AB-A3F872B2DB2D

**Type locality.** Kairei vent field (Monju Chimney), Central Indian Ridge, 25**°**19.2265′ S, 70°02.4181′ E, 2422 m in depth.

**Type materials**. Holotype (figure 3*a*): NSMT-Cr 26832, Kairei vent field (Monju Chimney), Central Indian Ridge, 25°19.2265′ S, 70°02.4181′ E, 2422 m in depth, collected by a slurp gun, HOV *Shinkai 6500* Dive #1175, R/V *Yokosuka* cruise YK09-13. Leg. 2 (principal scientist: Kentaro Nakamura), 13 November 2009, fixed and stored in 99.5% ethanol.

Paratypes. #1 (a cluster of 14 specimens; figure 2*a*), NSMT-Cr 26833, Kairei vent field (Monju Chimney), Central Indian Ridge, 25°19.2250′ S 70°02.4211′ E, 2426 m in depth, collected by a slurp gun in HOV *Shinkai 6500* Dive 1450, R/V *Yokosuka* YK16-E02 cruise (principal scientist: Ken Takai), 14 February 2016, fixed and stored in 10% seawater-buffered formalin. #2 (a cluster of seven specimens), same data as Paratype #1, fixed and stored in 99.5% ethanol (UMUT RA32760; figure 2*b*).

**Other materials examined**. One lot of three specimens (NSMT-Cr 26834; figure 3*b*), Solitaire vent field, Central Indian Ridge, 19°33.398′ S 65°50.871′ E, 2621 m in depth, HOV *Shinkai 6500* Dive #1327, R/V *Yokosuka* cruise YK13-02 (principal scientist: Manabu Nishizawa), 11 February 2013. One lot of five juvenile specimens (UMUT RA32761), same data as above. Further specimens used for measurements and DNA sequencing: nine specimens from Solitaire vent field (same data as above) and 10 specimens from Kairei vent field (same data as holotype or paratype #1).

**Diagnosis**. *Neolepas* with tridentoid mandibles. Second tooth without attached small elongated teeth. Tergum apex angle ranges from 60 to 65°.

**Description** (based on the holotype). Capitulum composed of eight fully calcified plates, including carina, rostrum, paired scutum, tergum, median latus (figures 3*a* and 5*a*). Peduncle to capitulum ratio 2 : 1 (figure 3*a*). All plates in capitulum with transverse growth ridges (figures 3*a* and 5). Tergum quadrangular with clear, sharp apical-basal ridge. Umbo apical, tergal apex angle 60° (figures 3 and 5). Basal angle of tergum located at capitulum-peduncle margin (figure 5*a*). Scutum quadrangular, tergal margin slightly concave, occludent margin slightly convex, apical-basal ridge slightly curved, scutum apex angle 33°, basal angle sharp, 56° (figure 5*a*). Median latus triangular, narrow, apex angle 30° (figure 5*a*). Height generally twice the width (figure 5*a*). Rostrum curved, scutal margin strongly curved, length of rostrum equal to height of median latus (figure 5*b*). Carina slightly curved, height of carina approximately 2/3 height of capitulum (figure 5*c*).

Peduncles with up to 26 peduncular scales per whorl, just below capitulum. Scales larger on the lower part of peduncle, becoming 15 per whorl by middle region of the peduncle. Scales approximately 0.7 mm wide, projecting 0.6 mm out of peduncle on lower region of peduncle.

Oral cone. Maxilla hatchet-shaped, margins with long simple setae, inferior angle protruded as blunt triangle (figure 6*a*). Simple setae present around margins of maxilla (figure 6*b*). Maxillule trapezoid, cutting edge straight with 24 large spines (figure 6*c*). Inferior and exterior margin with simple setae (figure 6*d*). Mandibles tridentoid, first tooth large and sharply pointed, cutting edges of second and third teeth denticulate (figure 6*e,f,g*). Lower margin and inferior angle with a number of spines (figure 6*h*). Dorsal view of mandibles reveals lack of small longitudinal teeth on second tooth (figure 7*a,b,c*) Mandibulatory palp elongated, with simple setae (figure 7*d*). Labrum cutting edge concaved, with one row of fine teeth (figure 7*e,f*).

Cirri. All six pairs of cirri are long and slender (figure 8). Cirral counts of anterior and posterior rami are given in table 1. Cirrus I, both anterior and posterior rami similar in length, protuberant at the last eight proximal segments (height approx. 3 times length), become antenniform starting from middle to distal region of ramus (figure 8*a*). Cirrus I bear simple type setae, setae become denser at proximal region of both rami (figure 9*a*). Cirrus II, anterior ramus and posterior ramus similar in length. Proximal 11 segments of both rami protuberant. Both rami become antenniform starting from middle to distal region of ramus (figure 8*b*). Setae in both rami simple (figure 9*b,c*). Proximal segments bear high density of setae (figure 8*b*). Intermediate segments of Cirrus II bear four pairs of long simple setae plus one pair of short simple setae (figure 9*d*) Cirri III to VI similar in morphology, both anterior and posterior rami similar in length. Intermediate segments of cirri III bear five pairs of long simple setae plus two pairs of short simple setae (figure 9*e*). Intermediate segments of cirri IV to VI bear five pairs of long simple setae plus one to two pairs of short simple setae (figure 9*f–h*). Length of long simple setae in cirri III to IV approximately 4–5 times length of an intermediate segment (figure 9). Caudal appendages unarticulate, short (figure 9*g*). Penis long, about half length of cirrus VI (figure 9*h*).

Juveniles. Some juvenile individuals were found attached on the lower part of the peduncle of the barnacles collected in the Solitaire vent field. Peduncle to capitulum ratio in five juveniles observed is about 1 : 1. The carina and rostrum in juveniles are relatively straight and apex extends beyond the margin of the capitulum (figure 3*c*).

**Etymology**. Latin, adjective (*maris* = sea; *indica* = Indian), named after its type locality and known distribution.

**Distribution**. Presently known from Kairei and Solitaire hydrothermal fields in the CIR (greater than 2500 m depth) and Longqi hydrothermal field in the SWIR (has also been referred to as the ‘Dragon vent field’ [6,16]). We consider the dredged material from 41° S site (site 21), SEIR [12] also represents the same species (see Discussion below).

*Remarks*: The present new species is placed in *Neolepas*, based on its ratio of rostrum to median latus (average 1.3 : 1 from Kairei and 1.45 : 1 from Solitaire populations) and approximately 20 peduncular scales per whorl. Presently, there are two other recognized species of *Neolepas*: *N. zevinae* and *N. rapanuii*. Although the present new species is clearly genetically distinct from both of these species, the genetic distance between *N. zevinae* and *N. rapanuii* seemed insufficient for separation at species level [6]. However, the capitular arrangement of these species exhibits difference, with the rostrum being as high as the median latus in *N. rapanuii*, about the same in *N. marisindica* sp. nov. and higher than the median latus in *N. zevinae* [22]. The main difference among these three species is seen in the morphology of the mandibles. *Neolepas zevinae* has a tridentoid mandible in which the second tooth of the mandible has elongated teeth (fig. 2*i* in [20]), *N. rapanuii* has a quadridentoid mandible with a small fourth tooth in-between the third tooth and the inferior margin (fig. 3*d* in [22]). In *N. marisindica* sp. nov., the mandible is tridentoid and without any small elongated teeth on the mandibular teeth. Among the three *Neolepas* species, the tergum of *N. marisindica* sp. nov. is the sharpest, having a mean apex angle of approximately 70°. The apex angles of both *N. rapanuii* and *N. zevinae* are approximately 75° [31].

**Morphological variations**. The Kairei field population specimens were with orange-coloured peduncle and the capitulum coated with dark brown mineral deposits (figures 3*a* and 4); the Solitaire field population was whitish and without mineral deposits (figure 3*b*). A specimen (6 K-1327-R2-1) from the Solitaire field was dissected to demonstrate the variation in the external morphology. Compared to the holotype, the specimen from Solitaire field had a wider tergal apex angle, at 73°. The ratio of rostrum to median latus was 1.3. Twelve peduncular scales present per whorl at the region below the capitulum. Scales were approximately 2.4 mm wide and projected 1.65 mm out of the peduncle (figure 3*b*).

Arthropodal characters from the Solitaire field specimens are similar to those of the holotype (Kairei field). Six pairs of cirri: cirral counts of both anterior and posterior rami of each cirrus are similar between the holotype and the Solitaire specimen concerned (figure 10 and table 1). Maxilla and maxillule of the specimen from the Solitaire field do not show great variation from the holotype (figure 11*a–d*). Both maxillule and maxilla with simple type setae. Mandibles tridentoid and without extra small elongated teeth on the second tooth (figures 11*d–h* and 12*a,b*). Mandibulatory palp elongated with simple setae (figure 12*c*), labrum with a single row of small teeth (figure 12*d–h*).

Comparing variations in capitular morphological characters between the Kairei population (10 specimens) and the Solitaire population (9 specimens), both populations shared similar peduncle characters: capitulum ratio (3 in Kairei and 3.8 in Solitaire), rostrum to median latus ratio (1.34 in Kairei and 1.45 in Solitaire), tergal apex angle (68 in Kairei and 71 in Solitaire) and the number of scales per whorl (20 in both populations; table 2). However, the Kairei specimens had significantly smaller scales (scale width 0.8 mm) when compared with the Solitaire population (1.54 mm; *t*-test, *t* = 3.5*,* d.f*.* = 17, *p* < 0.05; table 2, also figure 3*a,b*).

**Table 2.** Variation in morphological characters mean ± 1 s.d. (range) of *Neolepas marisindica* sp. nov. from Kairei and Solitaire hydrothermal fields.

	Kairei vents (*n* = 10)	Solitaire vents (*n* = 9)
capitulum height	15.8 ± 11 (6.9–25)	14.18 ± 5.8 (2.8–25)
peduncle: capitulum	3.08 ± 1.61 (1.7–6.0)	3.87 ± 1.94 (1.8–7.7)
R : ML	1.34 ± 0.25 (1.0–1.8)	1.45 ± 0.23 (1.1–1.7)
tergal apex angle	68.4 ± 6.1 (58–76)	71.3 ± 5.29 (66–81)
no. of scales per whorl	19.7 ± 4.7 (12–28)	20.3 ± 5.8 (14–30)
scale width	0.83 ± 0.14 (0.6–0.9)^a^	1.54 ± 0.32 (1.2–2.25)^a^
size of scales projected	0.83 ± 0.26 (0.5–1.16)	1.21 ± 0.2 (0.9–1.46)

^a^Indicates significant difference in *t*-tests, *p *< 0.05.

### 3.2. Molecular phylogenetic analysis

**Figure 13.**
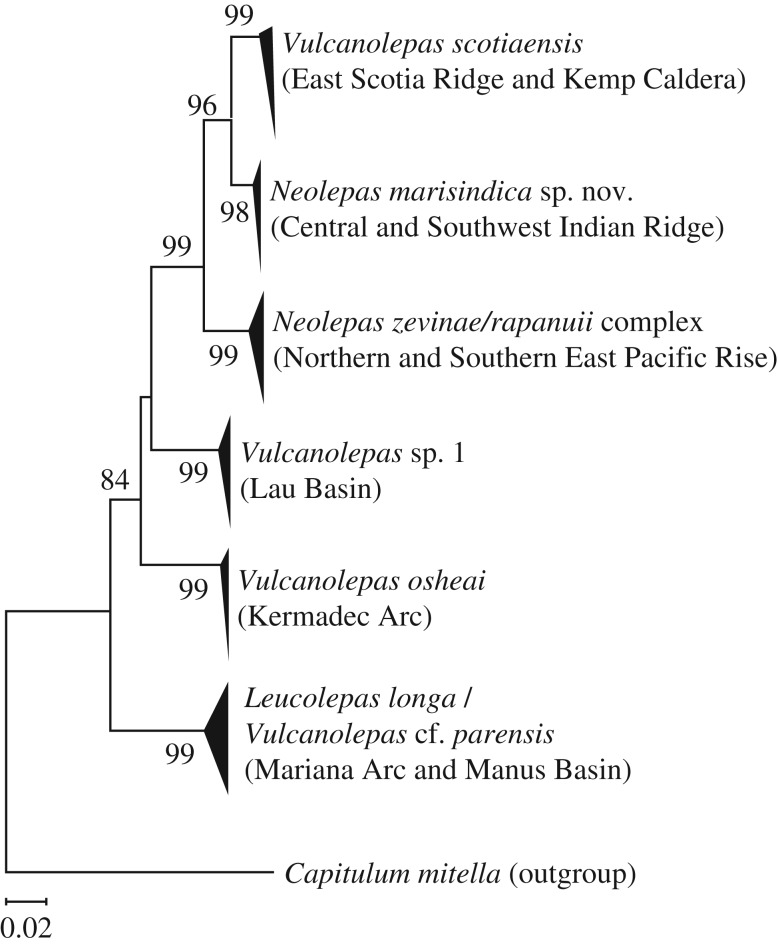
Molecular phylogenetic tree of eolepadid barnacles constructed by maximum-likelihood algorithm with Tamura three-parameter + gamma distribution model. Numbers beside each branch indicate bootstrapping value (2000 replicates, numbers lower than 70 not shown).

The reconstructed phylogenetic tree based on the maximum-likelihood algorithm is shown in figure 13. The relationships among eolepadid species were the same as previously shown [6], except for the additional OTUs of *Vulcanolepas* cf. *parensis* in Manus Basin [39], which was shown to share some haplotypes with *L. longa* in TOTO Caldera and Edison Seamount [6]. *Neolepas marisindica* sp. nov. from the three populations formed a single clade with previously reported sequences [6], which was sister to *V. scotiaensis* in the Southern Ocean. The *N. marisindica* sp. nov.–*V. scotiaensis* group is a sister group to EPR and Southern EPR populations of *N. zevinae-rapanuii* complex, whose outgroups consist of the undescribed *Vulcanolepas* species from the Lau Basin and the Tonga Arc [6] and *V. osheai* from the Kermadec Arc.

## 4. Discussion

### 4.1. Morphological variation and distribution range of *N. marisindica* sp. nov.

This study characterized *Neolepas marisindica* sp. nov. from deep-sea hydrothermal vent fields of the Indian Ocean, showing its morphological variability and phylogeography. As briefly mentioned above, the two CIR populations from Kairei and Solitaire hydrothermal vent fields of *Neolepas marisindica* sp. nov. examined in this study exhibited some differences in morphologies of capitulum and scales on peduncle, despite a lack of distinct sequence divergence in the COI gene between the two populations. The Solitaire hydrothermal field population, where diffuse flow venting was dominant, had larger scales with width greater than 1 mm compared with those from the Kairei hydrothermal field, where vigorous venting from black-smoker chimneys was dominant, whose scale width was approximately 0.8 mm. This difference was supported by statistical significance (*p* < 0.05; table 2). Morphological variations in neolepadines were also reported for *Vulcanolepas scotiaensis* in hydrothermal vent fields in the East Scotia Ridge, Southern Ocean, which exhibit a ‘robust’ form with short peduncle of peduncle : capitulum ratio as 1 : 1 in the site with low hydrothermal activity and a ‘gracile’ form with long peduncle of peduncle : capitulum ratio up to 20 : 1 in the site with active diffuse venting, but molecular analysis could not detect differences between the two [27]. The peduncular length is also variable in *Vulcanolepas parensis*, compared with the congeneric *V. osheai* and *L. longa* [27]. In the recent revision of taxonomy of Eolepadidae [27], the size of peduncular scales was used to discriminate *Vulcanolepas* and *Neolepas*, and the angle of tergal apex was considered diagnostic for *Leucolepas* and *Neolepas*. The presently examined specimens of *N. marisindica* sp. nov. exhibit intermediate characters between *Vulcanolepas* and *Leucolepas* in these two characters, respectively. The peduncular scales in the Kairei population are projected less than 1 mm out from the peduncle (table 2), which is within the diagnostic range indicated for *Vulcanolepas* [27]. Some individuals of *N. marisindica* sp. nov. had tergal apex angles of approximately 60°, which is supposedly a characteristic of *Leucolepas* (diagnostic tergal apex angle in *Neolepas* is 75° [27]).

The ratio of rostrum to median latus, as a key diagnostic character, is said to be 1.5 for *Neolepas* and 1 for other genera [27]. In this study, we found variations in the rostrum to median latus ratio among different specimens of *N. marisindica* sp. nov., which ranged from 1.0 to 1.8. The ratio of lengths of different capitular plates is clearly a continuous variable and it is highly problematic to treat these as the only diagnostic character for genus or even species identification, unless the range of variation is taken into consideration. We, therefore, suggest that the diagnostics and identification of *Neolepas* species is best relied upon investigation of arthropodal characters including mandibles, while also carefully considering their plate arrangement (with the intraspecific variation in mind), coupled with molecular DNA barcode analysis.

The present phylogenetic analysis was consistent with previous molecular studies [6,27], showing a close relationship between *V. scotiaensis* and members of the genus *Neolepas* (figure 13). This is different from taxonomic assignments based solely on hard part morphology, where *V. scotiaensis* was placed close to other *Vulcanolepas* species such as *V. osheai* [27]. These two species are then, in turn, sister to a clade consisting of *N. zevinae* and *N. rapanuii*, which appear to be genetically indistinguishable, at least using COI barcodes. This means *V. scotiaensis* is nested within the genus *Neolepas*. In addition, the mandible morphology of *V. scotiaensis* is actually very similar to those of other *Neolepas* species, as it has none or only minute longitudinal teeth. These results combined provide strong evidence that *V. scotiaensis*, in fact, belongs to the genus *Neolepas*, and therefore it is here formally transferred to *Neolepas*, as *Neolepas scotiaensis* (Buckeridge *et al*. [27]) comb. nov.

In contrast with high plasticity in the hard parts, morphologies of arthropodal characters are relatively stable and well supported by molecular phylogenetics. In this study, mandibles of the dissected individuals exhibited very similar morphological characteristics (figures 6*e*, 7*e*, 11*e*, 12*e*), whereas their hard part morphologies were more different (figure 3). The morphology of mandibles of *Neolepas* from SEIR [12] was the characteristic of *N. marisindica* sp. nov., as it lacks small longitudinal teeth on the second tooth. Therefore, we here consider these specimens to represent a further population of *N. marisindica* sp. nov., extending its distribution to SEIR, at least to 41° S. The DNA barcoding sequences of *Neolepas marisinsica* sp. nov. collected from Kairei and Solitaire hydrothermal fields on the CIR could not be separated from the Longqi population previously reported from the SWIR [6], confirming the distribution of the present new species on the SWIR, at least as far as the Longqi field. Therefore, *N. marisindica* sp. nov. is the only species of vent animal so far confirmed to range across hydrothermal vents in all three Indian Ocean oceanic ridges—the CIR, the SWIR and the SEIR. The fact that the same haplotypes have been recovered multiple times from populations on the CIR and the SWIR indicates that *N. marisindica* sp. nov. probably has sufficiently high dispersal ability to contain a metapopulation connecting the CIR and the SWIR across the Rodriguez Triple Junction, while for the scaly-foot gastropod *Chrysomallon squamiferum* the triple junction is known to act as a dispersal barrier [40]. As no vent on the SEIR has been visited by a submersible, further investigation of vents on the SEIR and samples from there will certainly reveal valuable information on the biogeography of deep-sea hydrothermal vent fauna in the Indian Ocean.

### 4.2. Phylogeography of vent barnacles

The phylogenetic analysis of vent stalked barnacles here elucidated their historical migration patterns across a geological timescale. As *V.* cf. *parensis* in Manus Basin shared some haplotypes with *L. longa* in TOTO Caldera and Edison Seamount (figure 13), here we regarded *V.* cf. *parensis* in Manus Basin as misidentification of *L. longa. Leucolepas longa* from the Mariana Forearc and the Manus Basin diversified at the most basal branch in the neolepadines, subsequently *Vulcanolepas osheai* in Kermadec Arc, and then an undescribed *Vulcanolepas* from the Lau Basin diversified, and finally the monophyletic *Neolepas* (figure 13). This renders *Vulcanolepas* paraphyletic. A previous tree published by Herrera *et al*. [6] combined three genes (28S, H3 and COI), however, with a different pattern with the basal branching being between a monophyletic *Neolepas* and a *Leucolepas*–*Vulcanolepas* clade. This node was highly supported in their study (0.88 and 100 for Bayesian posterior probability and bootstrap value, respectively). Considering that in our tree the node splitting *Leucolepas* from *Vulcanolepas–Neolepas* was not statistically supported (less than 0.70 in bootstrap probability), we interpret that the branching pattern observed in Herrera *et al*. [6] is more reliable. We, therefore, consider *Vulcanolepas* and *Neolepas* to be separate genera, following Herrera *et al.* [6]. On the other hand, the branching pattern within *Neolepas* (i.e. *N. zevinae/rapanuii* complex in the southern EPR, then *N. marisindica* sp. nov. in the Indian Ocean and finally *N. scotiaensis* in the Southern Ocean) was supported by high bootstrap probabilities (greater than 95 in bootstrap probabilities; figure 13).

The branching pattern, indicating close relationships between the species in Indian and Southern oceans compared with those in the southern EPR, is consistent with the pattern reported for the ‘yeti crabs’, squat lobsters in the genus *Kiwa* [7]. Neither *Neolepas* nor *Kiwa* has been reported from the Atlantic Ocean (except on the ESR of the Southern Ocean, which is technically in the extreme southern Atlantic), and their distribution and historical migration may be similar. The divergence between *N. marisindica* sp. nov. and *N. scotiaensis* was 1.7 Ma (95% HPD: 0.4–3.8) and the divergence between *N. marisindica* sp. nov.–*N. scotiaensis* and *N. zevinae*/*rapanuii* complex was 6.4 Ma (95% HPD: 3.0–11.2) [6]. The divergence between *Kiwa* sp. SWIR and *Kiwa tyleri* Thatje 2015 in Thatje *et al.* [41] from ESR, Southern Ocean was 1.5 Ma (95% HPD: 0.6–2.3) and the divergence between these two *Kiwa* species and *Kiwa hirsuta* from the Pacific-Antarctic Ridge was 19.1 Ma (95% HPD: 13.4–25.9) [7]. Geological evidence including the formation of the ESR and the Drake Passage, changes in the intensity and latitude of the Antarctic Circumpolar Current, and the realignment in the spreading axis of the Chile Rise seemed to have acted as key species vicariance events for *Kiwa* [7]. Characterization of faunal composition of hydrothermal vent fields on the ESR suggested the effect of the high intensity of the Antarctic Circumpolar Current around the middle Miocene (approx. 13.8 Ma) was crucial in separating vent fauna in ESR from other regions due to the inhibition of larval dispersal, as is known for non-vent Antarctic / Southern Ocean fauna [5,42]. Therefore, the present results strengthen the evidence to reject the hypothesis that Indian Ocean ridges act as ‘corridors’ for dispersal of vent taxa connecting Atlantic and Pacific Oceans [9], and instead, support the hypothesis of migration of vent fauna from the Pacific Ocean to the Indian Ocean, through the Southern Ocean. The phylogeny results indicate that the Pacific Ocean origin for the group may be the southern EPR, but this is necessarily only deduction from the known living species. Considering that a Jurassic neolepadine fossil (*?Neolepas augurata*) has been recorded from New Caledonia [25], it is highly likely that the true origin for the neolepadines is in the southwest Pacific near New Caledonia, from where it diversified towards both the EPR and Indian Ocean–Southern Ocean. Additionally, this is also in line with the fact that the basal taxa in Eolepadidae such as *Leucolepas longa* are found in the southwest Pacific.

In summary, we characterized a new hydrothermal vent barnacle *Neolepas marisindica* sp. nov. widely distributed in Indian Ocean vents which is hitherto the only species known to be distributed across all three mid-oceanic ridges in the Indian Ocean. Phylogeography of eolepadid stalked barnacles, including the new species, provides another piece of evidence against the Indian Ocean ‘corridor’ hypothesis. Future explorations of SEIR vents are urgently needed to shed further light on the biogeography of deep-sea hydrothermal vent taxa in the Indian Ocean and beyond. Morphological characteristics of hard parts in eolepadid barnacles, as in barnacles in general, were shown to exhibit high plasticity. We, therefore, suggest that the genus and species diagnostic characters should be mainly based on arthropodal characters (such as mouth parts) coupled with DNA barcoding, while the arrangement of hard parts such as the capitulum or scales on the peduncle should be used carefully with their intraspecific variation in mind.

## Supplementary Material

Table S1

## Supplementary Material

Phylogeography_Dataset

## Data accessibility

DNA sequences of *Neolepas marisindica* sp. nov. from Kairei and Solitaire hydrothermal vent fields on the Central Indian Ridge: GenBank accessions LC350007—LC350015. Datasets supporting this article have been uploaded as part of the electronic supplementary material, including electronic supplementary material, table S1 and the DNA sequence alignment in mega format.
